# Supplementary material for: Flexible and Printed Electrochemical Immunosensor Coated with Oxygen Plasma Treated SWCNTs for Histamine Detection
Source: Biosensors (Basel). 2020 Apr 10;10(4):35. doi: 10.3390/bios10040035 (PMC7235776; doi:10.3390/bios10040035)
Supplement: Supplementary file 1 [file biosensors-10-00035-s001.pdf]

biosensors

Supplementary Material

Flexible and Printed Electrochemical Immunosensor Coated with Oxygen Plasma treated SWCNTs for Histamine Detection

Bajramshahe Shkodra *, Biresaw Demelash Abera, Giuseppe Cantarella, Ali Douaki, Enrico Avancini, Luisa Petti * and Paolo Lugli

Faculty of Science and Technology, Free University of Bolzano-Bozen, 39100 Bolzano, Italy;
* Correspondence: Bajramshahe.Shkodra@natec.unibz.it (B.S.); Luisa.Petti@unibz.it (L.P.)

1. Immunosensor Development

The immunosensor realization was based on a direct enzyme-linked immunosorbent assays (ELISA) principle, where the antihistamine antibodies were placed onto the oxygen plasma OP treated single-walled carbon nanotubes (SWCNTs)-modified working electrode (WE). For the competitive reaction a mixture of 4 μL of the free His (diluted on PBS or fish extract) and 4 μL His-horseradish peroxidase (HRP) was placed onto the WE (Figure S1). The competitive reaction between the free His and the His-HRP to bind with the anti-histamine antibody was performed for 2 h at 37 °C.

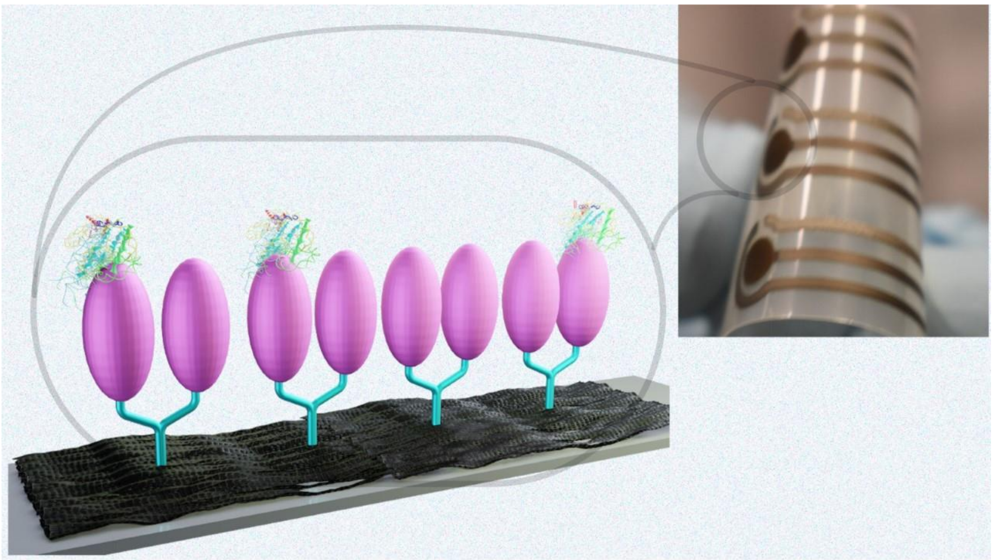

Figure S1. Scheme of His immunosensor developed on flexible screen-printed sensor.

In particular, the expected reaction (separately also qualitatively confirmed by an expected color change), is:

$$H_2O_2 + HRP_{(red)} \rightarrow HRP_{(ox)} + H_2O$$

$$HRP_{(ox)} + TMB_{(red)} \rightarrow HRP_{(red)} + TMB_{(ox)}$$

$$TMB_{(ox)} + 2H^{+} \rightarrow TMB_{(red)}$$

2. Electrode Thickness

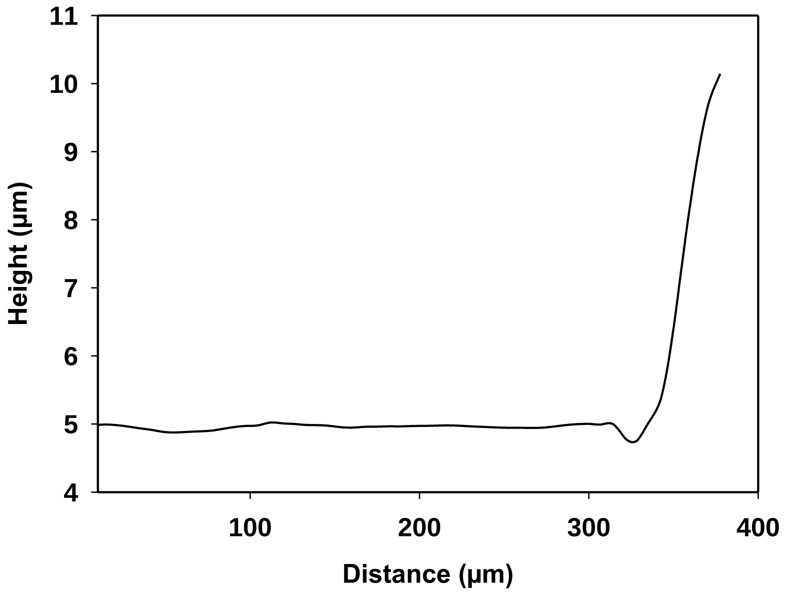

Figure S2. 2D profile of the silver electrode.

The electrode thickness was measured by a non-contact 3D-optical profilometer (ProFilm3D from Filmetrics, Unterhaching, Germany). The 2D profile for the thickness measurement is given in Figure SX, where the thickness was measured in terms of step height. The step height of the silver electrode was 5.38 µm.

3. Optimization of Spray Deposited SWCNTs Layers

To optimize the number of spray deposited layers on oxygen plasma (OP) treated silver electrode, cyclic voltammetry (CV) was performed. In this experiment, 50, 100, 150 and 200 SWCNTs layers were spray deposited on the OP-treated electrode, which were subsequently covered with 50 µl of 1 mM $[Fe(CN)_6]^{3-/4-}$ containing 0.1 M KCl. Finally, CV measurements were performed. It was found that 100 layers of spray deposited SWCNTs layers show the highest oxidation/reduction current, 45.61% enhancement on oxidation current and 76.12% enhancement on reduction current as compared to OP treated bare electrode. The oxidation/reduction current and the enhancement of the current generation as compared to the OP treated bare electrode are presented in Table S1.

Table S1. Oxidation and reduction current of cyclic voltammograms at a scan rate of 100 mV/s, in 1 mM $[Fe(CN)_6]^{3-/4-}$ containing 0.1 M KCl solution for different layers of SWCNTs.

Nr of SWCNTs layers	Oxidation Current	Reduction Current	Oxidation*/ Oxidation Bare	Reduction*/ Reduction Bare
Bare OP treated	1.03×10^{-2}	-1.23×10^{-2}	100.00%	100.00%
50	1.34×10^{-2}	-1.90×10^{-2}	129.40%	154.20%
100	1.51×10^{-2}	-2.17×10^{-2}	145.61%	176.12%
150	1.21×10^{-2}	-1.67×10^{-2}	117.43%	135.27%
200	1.46×10^{-2}	-1.94×10^{-2}	140.76%	157.62%

*Nr of layers of SWCNTs.

4. Fourier-Transform Infrared Spectroscopy Characterization of SWCNTs

Besides the higher current generation, the OP treatment leads to the formation of carbonyl and/or carboxylic groups as reported in the literature [1]. The presence of these groups can improve the immobilization of antibodies on the surface of SWCNTs. To confirm the presence of these polar

groups we have analyzed the SWCNTs before and after OP treatment using a FT-IR INVENIO-R e ATR spectrophotometer (Bruker), operating in transmittance mode to record the spectra over 400–4000 cm^{-1}. Figure S2 shows Fourier-transform infrared (FTIR) spectra of a) spray deposited SWCNTs and b) spray deposited SWCNTs treated with OP. In both spectra, the peaks at 2916 and 2849 cm^{-1} could be assigned to C-H stretching vibrations, while C=C bending vibrations are observed at 983 and 831 cm^{-1} [2]. A broad peak at about 3460 cm^{-1} could be assigned to the O–H stretch, while the peak at 1217 cm^{-1} can be associated with C–O stretching vibrations, the intensity of the peaks is much higher after OP treatment which indicates the OP treatment led to the graft of polar groups [3]. Moreover, the peak at 1472 cm^{-1} can be associated with the C-O-H stretching [2]. FTIR results indicate the presence of O–H, C–O stretching vibrations rather than carbonyl groups could be the consequence of different initial surface conditions and Different OP parameters.

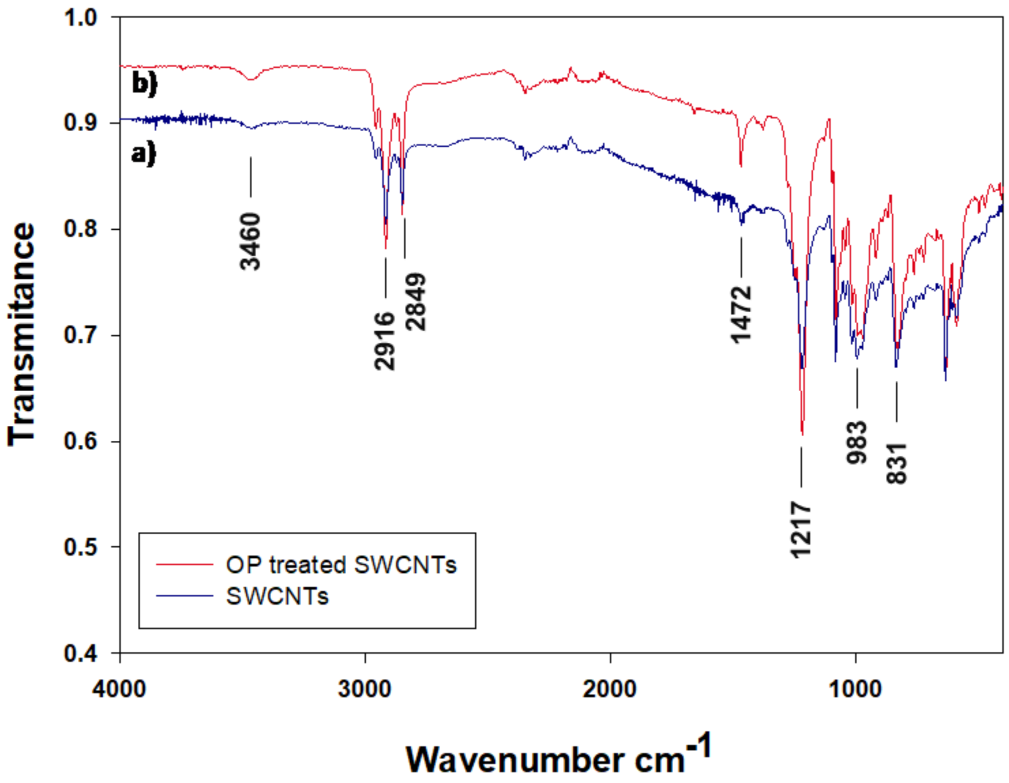

Figure S3. Fourier-transform infrared spectra of (**a**) spray deposited SWCNTs and (**b**) spray deposited SWCNTs treated with OP.

5. Flexibility, Regeneration and Time Stability of Histamine Immunosensor

After the first regeneration and 100 bending cycles the current generation was 100.71% with a standard deviation of ±0.60 μA. After the second regeneration and 250 bending cycles the current generation was 94.66% with a standard deviation of ±0.78 μA. After third regeneration and 500 bending cycles the current generation was 77.97% with a standard deviation of ±0.96 μA. After the fourth generation and 1000 bending cycles the current generation was 52.85% with a standard deviation of ±2.51 μA.

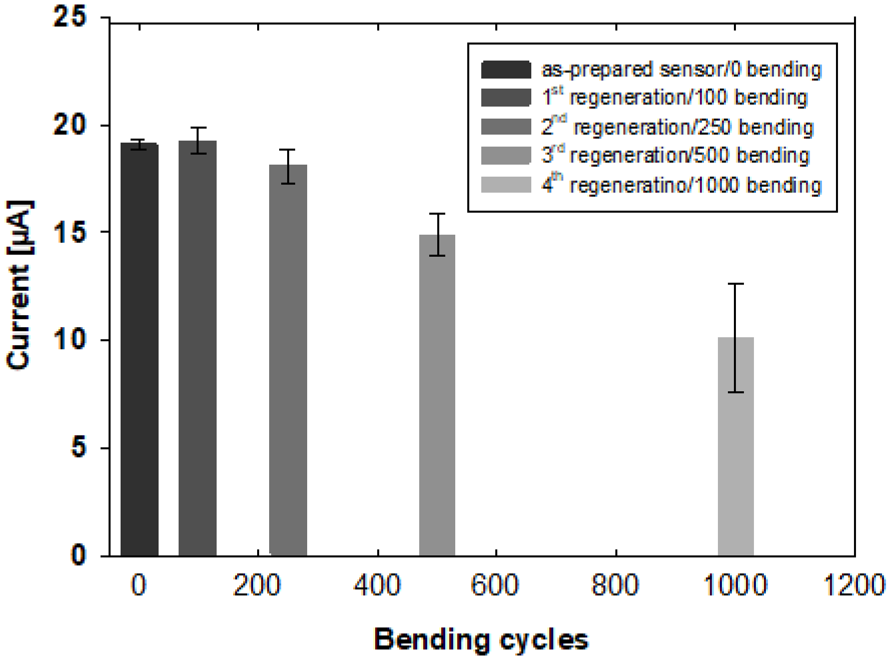

Figure S4. Regeneration and bending of the histamine immunosensor. Error bars estimated as a triple of the standard deviation (n = 3).

Every 2 days three sensors already prepared were tested to quantify 5 ng/mL of His diluted-on PBS pH = 7.4 for one month. After 12 days, the current generation was 95.29% of its initial value (as displayed in Figure S4).

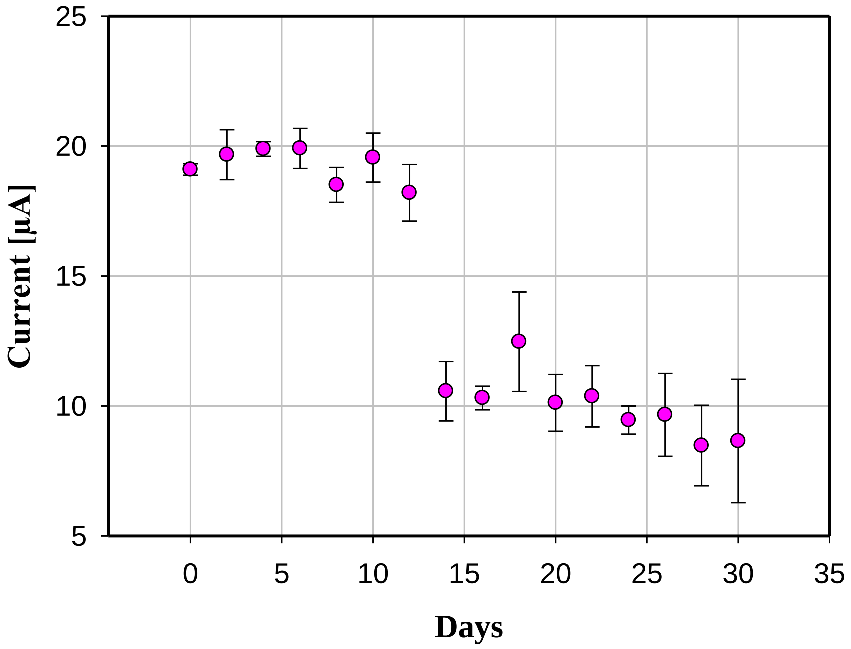

Figure S5. Time stability test for histamine immunosensor. Detection of 5 ng/mL of His every 2 days for one month. Error bars estimated as a triple of the standard deviation (n = 3).

References

1. Chetty, R.; Maniam, K.K. Schuhmann, W.; Muhler, M. Oxygen-plasma-functionalized carbon nanotubes as supports for platinum-ruthenium catalysts applied in electrochemical methanol

oxidation. *Chempluschem* **2015,** *80*, 130–135.

2. Atieh, M.A.; Bakather, O.; Al-Tawbini, Y.B.; Bukhari, A.A. Abuilaiwi, F.A.; Fettouhi, M.B. Effect of carboxylic functional group functionalized on carbon nanotubes surface on the removal of lead from water *Bioinorg. Chem. Appl.* **2010**, *2010,* 1–9.
3. Nguyen, V.H.; Shim, J.J. Green synthesis and characterization of carbon nanotubes/polyaniline nanocomposites. *J. Spectrosc.* **2015**, *2015,* 1–9.

© 2020 by the authors. Licensee MDPI, Basel, Switzerland. This article is an open access article distributed under the terms and conditions of the Creative Commons Attribution (CC BY) license (http://creativecommons.org/licenses/by/4.0/).
